# PP121, a dual inhibitor of tyrosine and phosphoinositide kinases, relieves airway hyperresponsiveness, mucus hypersecretion and inflammation in a murine asthma model

**DOI:** 10.1186/s10020-023-00748-w

**Published:** 2023-11-07

**Authors:** Wei Li, Lu Xue, Changsi Peng, Ping Zhao, Yongbo Peng, Weiwei Chen, Wenyi Wang, Jinhua Shen

**Affiliations:** Institute for Medical Biology and Hubei Provincial Key Laboratory for Protection and Application of Special Plants in Wuling Area of China, College of Life Sciences, South-Central Minzu University, Wuhan, 430074 China

**Keywords:** PP121, Asthma, Airway hyperresponsiveness, Mucus secretion, Inflammation

## Abstract

**Background:**

Tyrosine kinase and phosphoinositide kinase pathways play important roles in asthma formation. As a dual tyrosine and phosphoinositide kinase inhibitor, PP121 has shown anticancer efficacy in multiple tumors. However, the study of PP121 in pulmonary diseases is still limited. Herein, we investigated the therapeutic activities of PP121 in asthma treatment.

**Methods:**

Tension measurements and patch clamp recordings were made to investigate the anticontractile characteristics of PP121 in vitro. Then, an asthma mouse model was established to further explore the therapeutic characteristics of PP121 via measurement of respiratory system resistance, histological analysis and western blotting.

**Results:**

We discovered that PP121 could relax precontracted mouse tracheal rings (mTRs) by blocking certain ion channels, including L-type voltage-dependent Ca^2+^ channels (L-VDCCs), nonselective cation channels (NSCCs), transient receptor potential channels (TRPCs), Na^+^/Ca^2+^ exchangers (NCXs) and K^+^ channels, and accelerating calcium mobilization. Furthermore, PP121 relieved asthmatic pathological features, including airway hyperresponsiveness, systematic inflammation and mucus secretion, via downregulation of inflammatory factors, mucins and the mitogen-activated protein kinase (MAPK)/Akt signaling pathway in asthmatic mice.

**Conclusion:**

In summary, PP121 exerts dual anti-contractile and anti-inflammatory effects in asthma treatment, which suggests that PP121 might be a promising therapeutic compound and shed new light on asthma therapy.

**Supplementary Information:**

The online version contains supplementary material available at 10.1186/s10020-023-00748-w.

## Keypoints

1. PP121 has anticontractile properties in precontracted mTRs.
2. PP121 inhibits calcium mobilization by switching certain ion channels.
3. PP121 relieves typical pathological signs in an asthma mouse model.
4. PP121 downregulates inflammatory factors, mucins and MAPK/Akt signaling.

**Supplementary Information:**

The online version contains supplementary material available at 10.1186/s10020-023-00748-w.

## Introduction

Worldwide, asthma is one of the most common respiratory diseases in both children and adults (Agache et al. 2021; Ioniuc, et al. 2022; Cruz et al. 2023; Baker and Houin 2023). As a chronic respiratory condition, asthma is mainly characterized by airway inflammation, remodeling, and abnormal contraction (Casaro et al. 2019; Lee and McDonald 2018; Patadia et al. 2014; Brand et al. 2015). Inhaled bronchodilators, especially β2-agonists, are at the forefront of asthma treatment to relieve a series of airway disorders. However, patients might quickly develop resistance to the current bronchodilators (Ashton and Hancox 2018; Suissa et al. 2017; Ayed et al. 2020; Salmon et al. 2014). Therefore, the investigation of new drugs for asthma treatment is necessary.

Tyrosine kinase signaling and phosphoinositide kinase signaling are crucial signaling cascades in the pathogenesis of asthma (Tundwal and Alam 2012; Wong 2005; Yoo et al. 2017). Tyrosine kinase inhibitors (TKIs) and phosphoinositide kinase inhibitors (PKIs) have been identified to have anti-inflammatory and antitumor effects in lung diseases, especially asthma (Murugesan et al. 2021; Ohmori, et al. 2021; Moran-Mendoza et al. 2019; Lamb 2021; Stokes and Condliffe 2018; Tsay et al. 2018; Duan et al. 2005), which shed light on the application of TKIs and PKIs in asthma treatments. As a small-molecule inhibitor, PP121 was first identified from the systematic discovery of a novel dual inhibitor of tyrosine and phosphoinositide kinases (Apsel et al. 2008). Recent studies have revealed that PP121 participates in the regulation of many cancer cell processes, including cell survival and cell motility. For example, PP121 could inhibit cell growth and migration in anaplastic thyroid carcinoma (Che et al. 2014). PP121 also exerts cytotoxic effects in human esophageal cancer cells via Akt/mammalian target of rapamycin (mTOR) and nuclear factor kappa-B (NFκB) signaling (Peng et al. 2015). As a TKI, PP121 also participates in the treatment of prostate cancer (Bello, et al. 2021) and hepatocellular carcinoma (Zhai et al. 2021). In addition to antitumor activities, PP121 plays potential roles in osteolytic diseases (Zhou et al. 2020). However, the study of PP121 in lung diseases, especially asthma, is still limited.

In view of this, the current study aimed to investigate the potential therapeutic activity of PP121 in asthma treatment. A series of electrophysiological experiments and animal experiments were designed to explore the effect of PP121 on airway hyperresponsiveness and airway inflammation, which are two main symptoms of asthma. We found that PP121 could relax precontracted mTRs by regulating calcium mobilization and blocking certain ion channels, including L-VDCCs, NSCCS, TRPCs, NCXs and K^+^ channels, especially large-conductance Ca^2+^-activated K^+^ channels (BK channels). Moreover, PP121 relieved pathological features, including airway hyperresponsiveness, airway narrowing, systematic inflammation and mucus secretion, in asthmatic mice. The investigation of potential molecular mechanisms showed that PP121 could downregulate vascular endothelial growth factor (VEGF), vascular endothelial growth factor receptor 2 (VEGFR2), mucin 5 subtype AC (MUC5AC), mucin 5 subtype B (MUC5B), tumor necrosis factor alpha (TNF-α), interleukin 4 (IL-4) and interleukin 5 (IL-5). Furthermore, PP121 could suppress the MAPK/Akt signaling pathway by reducing the phosphorylation of MAPK and Akt. Thus, both the anticontractile and anti-inflammatory properties of PP121 suggest that PP121 can be used as a potential therapeutic agent for asthma.

## Materials and methods

### Reagents and chemicals

Gadolinium (G7532-5G), pyrazole 3 (Pyr3, P0032-5MG), tetraethylammonium chloride (TEA-Cl, T2265-100G), nifedipine (C11875500BT), niflumic acid (NA, N0613-10G) and paxilline (PAX, P2928-10MG) were purchased from Sigma (St. Louis, MO, USA). Dexamethasone (Dex, MB1434) was purchased from Meilunbio (Dalian, China). PP121 (V0200) was purchased from InvivoChem (Guangzhou, China). Acetylcholine chloride (ACh, S30170-5 g), and ovalbumin (OVA, S28623-25 mg) were purchased from Yuanye Bio-Technology (Shanghai, China), and 3-(4,5-dimethylthiazol-2-yl)-2,5-diphenyltetrazolium bromide (MTT, G4000) was purchased from Promega (Beijing, China). All chemicals were purchased from Sinopharm Chemical Reagent Co. (Shanghai, China) unless stated otherwise. The antibody for β-actin (AA128) was purchased from Beyotime (Shanghai, China). The antibodies for p-VEGFR2 (AP0382) and VEGFR2 (A11127) were purchased from ABclonal (Wuhan, China). Antibodies against p-Akt (#4058S), Akt (#4685S), p-p38 MAPK (#4631S) and p38 MAPK (#9212) were purchased from Cell Signaling Technology (Beverly, MA, USA). A human bronchial epithelial cell line (16HBE, ZQ0001) was purchased from Zhongqiaoxinzhou (Shanghai, China).

### Establishment of an asthmatic mouse model

The establishment of the asthma mouse model was performed as previously described (Shi et al. 2020). Briefly, male BALB/c mice (6–8 weeks old, sexually mature, Zikeheng Biotech, Wuhan, China) were housed in the Experimental Animal Center of South-Central Minzu University, which provided a specific pathogen-free (SPF)-grade environment. All animal procedures were designed and performed under the supervision of the Animal Care and Ethics Committee of South-Central Minzu University. Briefly, sexually mature mice were randomly divided into a control group (n = 6 mice), an asthma group (n = 6 mice), a Dex group (n = 6 mice) and a PP121 group (n = 6 mice). For establishment of the asthma mouse models, the asthma group, Dex group and PP121 group were OVA-sensitized by intraperitoneal (IP) injections of 3 mg/mL OVA (200 µL per 20 g) on Days 0, 7 and 14, while the control group was injected with physiological saline. From Day 21 to Day 34, the asthma group, Dex group and PP121 group were stimulated with the intranasal instillation of 3 mg/mL OVA (20 μL per 20 g, once per day), supplemented with daily gavage of Dex (3 mg/kg) in the Dex group or PP121 (20 mg/kg and 50 mg/kg, respectively) in the PP121 group. The control group was intranasally instilled and gavaged with physiological saline.

### Measurement of mouse airway smooth muscle tension

The measurement of mouse airway smooth muscle tension was performed as previously described with subtle modifications (Luo, et al. 2018). Tracheae (n = 6/6 mice) were dissected from euthanized mice and transferred to ice-cold physiological salt solution (PSS: 2 mM CaCl_2_, 10 mM glucose, 10 mM HEPES, 5 mM KCl, 1 mM MgCl_2_·6H_2_O, 135 mM NaCl, pH adjusted to 7.4 with NaOH). Then, 5–7 mm mTRs were isolated and mounted in a 6 mL organ bath (Techman, Chengdu, China) filled with PSS bubbled with 95% O_2_ and 5% CO_2_ at 37 °C. The mTRs were equilibrated for 60 min, and then, the experiments were conducted with 80 mM K^+^ or 100 μM ACh. For analysis of the participation of NCXs, PSS was replaced with Li-PSS (2 mM CaCl_2_, 10 mM glucose, 10 mM HEPES, 5 mM KCl, 135 mM LiCl, 1 mM MgCl_2_·6H_2_O, pH adjusted to 7.4 with Tris-base).

For the cross-sectional area measurement of the bronchus, tracheae and lungs (n = 6/6 mice) were isolated from euthanized mice. The lung tissues were filled with 41 °C low melting agarose through the cannulated tracheae. Then, the tracheae and lungs were transferred to Hanks’ balanced salt solution (HBSS: 1.26 mM CaCl_2_, 5.56 mM glucose, 20 mM HEPES, 5.33 mM KCl, 0.44 mM KH_2_PO_4_, 0.49 mM MgCl_2_·6H_2_O, 0.41 mM MgSO_4_, 0.34 mM Na_2_HPO_4_, 137.93 mM NaCl, 4.17 mM NaHCO_3_, pH adjusted to 7.4 with NaOH) and incubated on ice for 30 min at 4 °C. The agarose-inflated lungs were sectioned into 350 μm thick slices with a vibratome (VT1000S, Leica, Nussloch, Germany). The lung slices were incubated in HBSS, and the lumen area of the bronchus under different stimuli was photographed and analyzed with LightTools (Optical Research Associates, CA, USA).

### Measurement of channel currents

For isolation of single mouse airway smooth muscle cells (mASMCs) for current measurement, the mouse tracheal muscles were removed from the trachea in mASMC dissociation buffer (0.1 mM CaCl_2_, 11 mM glucose, 10 mM HEPES, 5.2 mM KCl, 0.6 mM KH_2_PO_4_, 1.2 mM MgCl_2_, 120 mM NaCl, and 25 mM NaHCO_3_, pH adjusted to 7.0 with NaOH). The dissected smooth muscles were incubated and digested for 22–24 min at 37 °C in digest solution I, which contained 2–3 mg/mL papain, 1 mg/mL bovine serum albumin (BSA), and 0.15 mg/mL dithiothreitol (DTT), and then dissolved in mASMC dissociation buffer. Then, the tissues were transferred to digest solution II (1 mg/mL collagenase H, 1 mg/mL BSA, dissolved in mASMC dissociation buffer) for 3–5 min at 37 °C. The tissues were washed with 1 mg/mL BSA and gently flicked to yield single mASMCs. mASMCs were kept on ice for further experiments.

The measurement of channel currents was conducted as previously described (Shi et al. 2020). For measurement of L-VDCC currents, mASMCs (n = 5 cells/5 mice) were patched and held in bath solution I (27.5 mM BaCl_2_, 10 mM HEPES, 11 mM glucose, 1 mM MgCl_2_, 107 mM NaCl, 10 mM TEA-Cl, pH adjusted to 7.2 with NaOH) at − 70 mV. The pipette was filled with intracellular solution I (130 mM CsCl, 10 mM EGTA, 10 mM HEPES, 4 mM MgATP, 4 mM MgCl_2_, pH adjusted to 7.3 with CsOH). L-VDCC currents were recorded with an EPC-10 patch-clamp amplifier (HEKA, Lambrecht, Germany) under a stepped voltage ranging from − 70 to + 40 mV in 10 mV increments every 50 ms.

For measurement of NSCC currents, mASMCs (n = 5 cells/5 mice) were patched and held in bath solution II (1.5 mM CaCl_2_, 11 mM glucose, 10 mM HEPES, 126 mM NaCl, pH adjusted to 7.2 with NaOH) at − 60 mV. The pipette was filled with intracellular solution II (1 mM CaCl_2_, 108 mM cesium acetate, 18 mM CsCl, 3 mM EGTA, 10 mM HEPES, 1.2 mM MgCl_2_, pH adjusted to 7.2 with Tris-base). NSCC currents were recorded with a 500 ms ramp from − 80 to + 60 mV, and the data at 70 mV were used to construct current–time curves.

For measurement of BK_Ca_ channel currents, mASMCs (n = 5 cells/5 mice) were patched and held in bath solution III (5.4 mM CaCl_2_, 10 mM HEPES, 5.4 mM KCl, 0.8 mM MgCl_2_, 150 mM NaCl, pH adjusted to 7.2 with KOH). The pipette was filled with intracellular solution III (10 mM EGTA, 10 mM HEPES, 125 mM KCl, 6.2 mM MgCl_2_, 10 mM NaCl, pH adjusted to 7.2 with KOH). BK currents were recorded from a holding potential of − 80 to + 80 mV in 10 mV increments.

### Measurement of cell viability in 16HBE cells

MTT assays were performed on 16HBE cells to analyze the effect of PP121 on cell viability. Approximately 2 × 10^3^ 16HBE cells (per well) were seeded in 96-well plates with 3.16 μM, 10 μM and 56.23 μM PP121. After 24 h or 48 h of incubation, 100 µg MTT reagent was added to each well and incubated for 4 h. Then, 150 µL of dimethyl sulfoxide (DMSO) was added, and the absorbance at 490 nm was recorded.

### Measurement of respiratory system resistance

The respiratory system resistance (Rrs) was measured with a forced oscillation technique as previously described (Shi et al. 2020; Chen et al. 2019). In brief, mice were anesthetized with an intraperitoneal injection of 1% sodium pentobarbital (10 mg/kg). Then, the anesthetized mouse was tracheostomized and connected with the flexiVent system (SCIREQ, Montreal, PQ, Canada). Aerosolized ACh was gradually added to the system at increasing doses (3.125, 6.25, 12.5, 25 and 50 mg/mL). Then, dose‒response curves were generated, and the results were analyzed in Flexiware 8 software (SCIREQ, Montreal, PQ, Canada).

### Histological analysis

Trachea and left lung specimens from 3 mice per group (control, asthma, Dex and PP121) were dissected and fixed in 4% paraformaldehyde (PFA, Servicebio, Wuhan, China). Hematoxylin and eosin (H&E) and periodic acid-Schiff (PAS) staining were conducted by Servicebio, Wuhan, China. Stained sections (3 slides/3 mice) were photographed for further analysis.

### Reverse transcription and quantitative real-time PCR

The right lung specimens of mice were cut into small pieces (< 1 mm^3^) and then homogenized in TRIzol^®^ reagent with a hand-held homogenizer. Total RNA was extracted with an RNA extraction kit (TaKaRa, Otsu, Japan), and template cDNA was synthesized with a cDNA synthesis kit (TaKaRa, Otsu, Japan) according to the manufacturer’s protocol. The primer sequences were as follows:

Actin-F: 5′-AGAGGGAAATCGTGCGTGAC-3′

Actin-R: 5′-CAATAGTGATGACCTGGCCGT-3′

IL-4-F: 5′-AACGAAGAACACCACAGAGAGTG-3′

IL-4-R: 5′-CGATGAATCCAGGCATCGAAAAG-3′

IL-5-F: 5′-CGCTCACCGAGCTCTGTTG-3′

IL-5-R: 5′-CCAATGCATAGCTGGTGATTTTT-3′

MUC5AC-F: 5′-ATGGGCTGTGTTCCTGTGTC-3′

MUC5AC-R: 5′-CAGAACATGTGTTGGTGTGCAGTC-3′

MUC5B-F: 5′-GTGAGGAGGACTCCTGTCAAGT-3′

MUC5B-R: 5′-CCTCGCAGAAGGTGATGTTG-3′

TNF-ɑ-F: 5′-TGGAAGACTCCTCCCAGGTA-3′.

TNF-ɑ-R: 5′-ACGGCATGGATCTCAAAGAC-3′.

VEGF-F: 5′-ATGGATGTCTACCAGCGAAGCTACTG-3′

VEGF-R: 5′-GGTTTGATCCGCATGATCTGCA-3′

VEGFR2-F: 5′-CACCTGCCAGGCCTGCAA-3′

VEGFR2-R: 5′-GCTTGGTGCAGGCGCCTA-3′

Real-time PCR and melting curve analysis were carried out with SYBR Green qPCR Mix (Biosharp, Hefei, China) using the Applied Biosystems 7500 Fast Real-Time PCR System® (Applied Biosystems, Foster City, CA, USA) with the default program according to the manufacturer’s instructions. The mRNA expression levels of related genes were calculated utilizing the 2^−ΔΔCt^ method and normalized to the expression levels of actin.

### Western blotting

The lung tissues of mice were homogenized and lysed for 30 min in ice-cold RIPA buffer (Beyotime, Shanghai, China) with 1% PMSF (Beyotime, Shanghai, China). Then, the lysates were centrifuged at 16,000 ×*g* for 15 min at 4 °C. The supernatant was collected and quantified. Total proteins (20 μg) were resolved by sodium dodecyl sulfate–polyacrylamide gel electrophoresis (SDS-PAGE) and transferred to nitrocellulose (NC) membranes. The membrane was blocked in Tris-buffered saline tween-20 (TBST, 8 g/L NaCl, 2.42 g/L Tris-base, 0.1% Tween 20) with 5% (wt/vol) powdered milk for 90 min and then incubated with primary antibodies (1:1000 dilution) at 4 °C overnight. After 3 washes with TBST, the proteins were blotted with horseradish peroxidase (HRP)-goat anti-rabbit IgG (CW0103S, CWBIO, Beijing, China) or HRP-goat anti-mouse IgG (EO32210-02, EARTHOX, Burlingame, CA, USA). The signal was detected by an ECL plus kit (Yeasen, Shanghai, China) and analyzed with Image Lab 3.0 (Bio-Rad, Hercules, CA, USA) according to the manufacturer’s protocol. β-Actin was used as an internal control.

### Statistical analysis

All the experimental results were analyzed using Student’s *t* test with Origin 8.0 (OriginLab, Northampton, MA, USA). All statistical data are presented as the means ± standard deviations (SD). *p* < 0.05 was considered statistically significant.

## Results

### PP121 relaxed precontracted mTRs in a dose-dependent mechanism

As a dual inhibitor of tyrosine and phosphoinositide kinases, PP121 plays important roles in multiple diseases (Che et al. 2014; Peng et al. 2015; Bello, et al. 2021; Zhou et al. 2020). However, the therapeutic function of PP121 in pulmonary diseases, especially asthma, is still unclear. A recent study revealed that inhibitors of receptor tyrosine kinases (RTKs) or phosphatidylinositol-3-kinase (PI3K) could improve lung function in asthma (Kim et al. 2021; Bi et al. 2020). Herein, we intend to explore the potential therapeutic role of PP121 in asthma, especially airway hyperresponsiveness and airway inflammation.

**Fig. 1:**
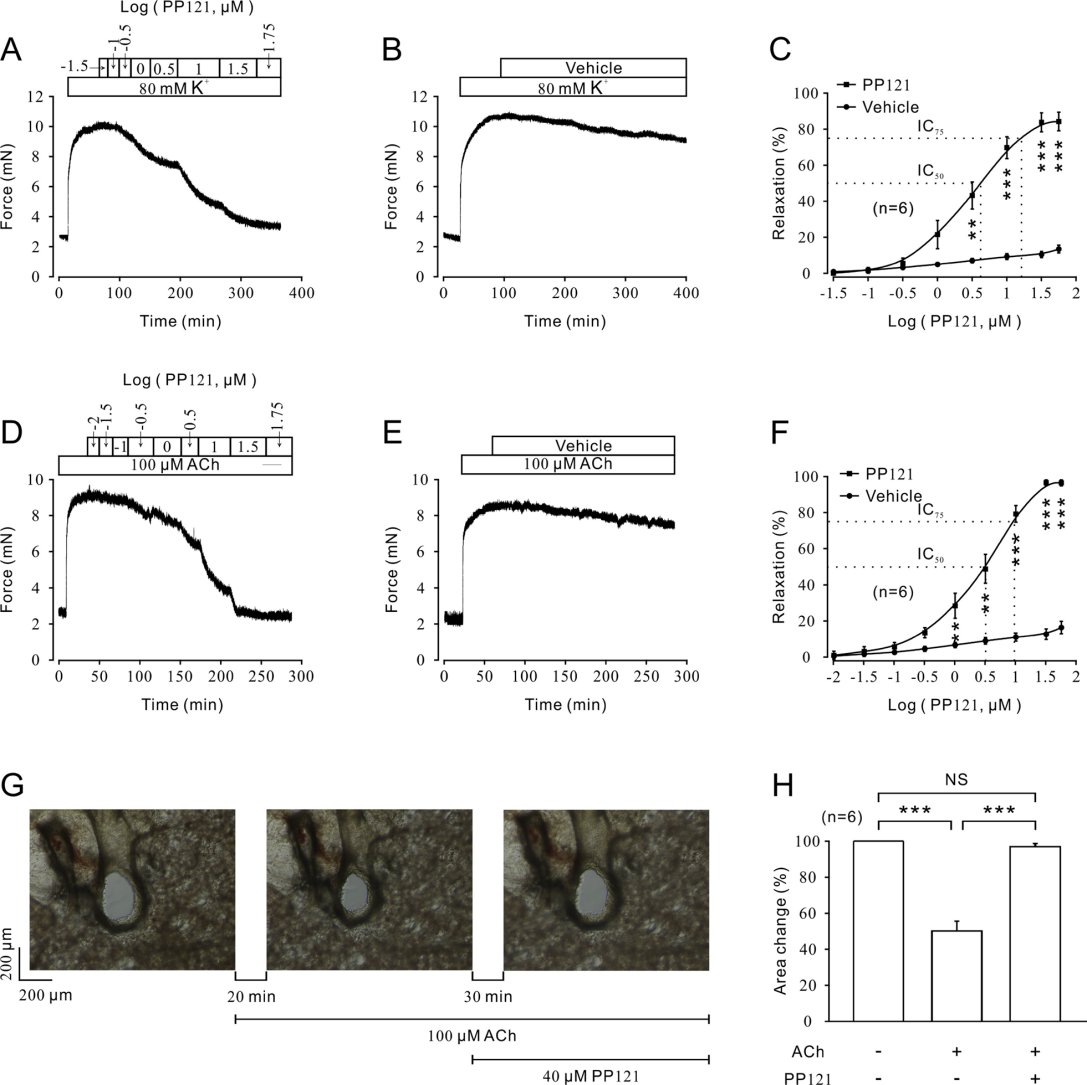
PP121 relaxed high K^+^ or ACh-precontracted mTRs in a dose-dependent manner. **A** PP121 inhibited 80 mM K^+^-induced precontraction in a dose-dependent manner. **B** The DMSO solution (vehicle) failed to induce relaxation compared with (**A**). **C** The dose-relaxation curves of PP121 or vehicle are presented (n = 6/6 mice). **D** PP121 relaxed 100 µM ACh-induced precontraction in a dose-dependent manner, while vehicle could not induce relaxation under the same conditions (**E**). **F** The dose-relaxation curves of PP121 or vehicle are presented (n = 6/6 mice). **G** PP121 (40 µM) significantly relieved 100 µM ACh-induced bronchial cross-sectional area narrowing, while the bar graph is presented in (**H**) (n = 6/6 mice). *p < 0.05; **p < 0.01; ***p < 0.001

For analysis of the potential relaxant role of PP121 on abnormal airway muscle contraction, 80 mM K^+^ or 100 μM ACh was employed to induce a precontraction of mTRs (Fig. 1). As shown in Fig. 1A, 80 mM K^+^ could induce a steady contraction, which could be gradually inhibited by PP121 in a dose-dependent manner (Fig. 1A). Moreover, the dissolved DMSO solution had no relaxant effect on precontracted mTRs, which excluded the potential disturbance of the vehicle. The dose-contraction curve for Fig. 1A is shown in Fig. 1C. The half-maximal inhibitory concentration (IC_50_) was 4.19 ± 0.06 µM, while the IC_75_ was 14.98 ± 0.06 µM. The maximal relaxation was 84.92 ± 0.63%.

In addition to 80 mM K^+^, 100 μM ACh, an important agonist of ion channels, was used to precontract mTRs. As shown in Fig. 1D, 100 μM ACh induced a similar steady contraction compared with 80 mM K^+^, and PP121 could also reverse the precontraction in a dose-dependent manner. The DMSO solution had no effect on the precontraction evoked by 100 µM ACh, which is similar to Fig. 1A. Furthermore, according to the concentration-relaxation curve shown in Fig. 1F, the IC_50_ and IC_75_ were 2.77 ± 0.06 µM and 8.62 ± 0.06 µM, respectively. The maximal relaxation was 99.15 ± 1.17%. Further cross-sectional area measurements of the bronchus showed that 40 µM PP121 could reverse 100 μM ACh-induced bronchus narrowing (Fig. 1G, H). To exclude the possible side effects of PP121 on mTRs, we determined the effect of PP121 on tissue bioactivity. As shown in Additional file 1: Fig. S1A, 56 μM PP121 had no effect on resting mTRs. Further tension measurements showed that 56 μM PP121 could erase 80 mM K^+^-induced contraction. After washout, 80 mM K^+^ evoked a similar contraction (Additional file 1: Fig. S1B, C). Moreover, 100 μM ACh-induced precontraction could be inhibited by 40 μM PP121. After washout, a similar contraction was evoked (Additional file 1: Fig. S1D, E). These results indicated that the relaxant effect of PP121 on mTRs is reversible. Thus, PP121 exerted a relaxant effect on airway muscle and showed limited harm to tissue bioactivity.

### PP121 altered calcium mobilization

**Fig. 2:**
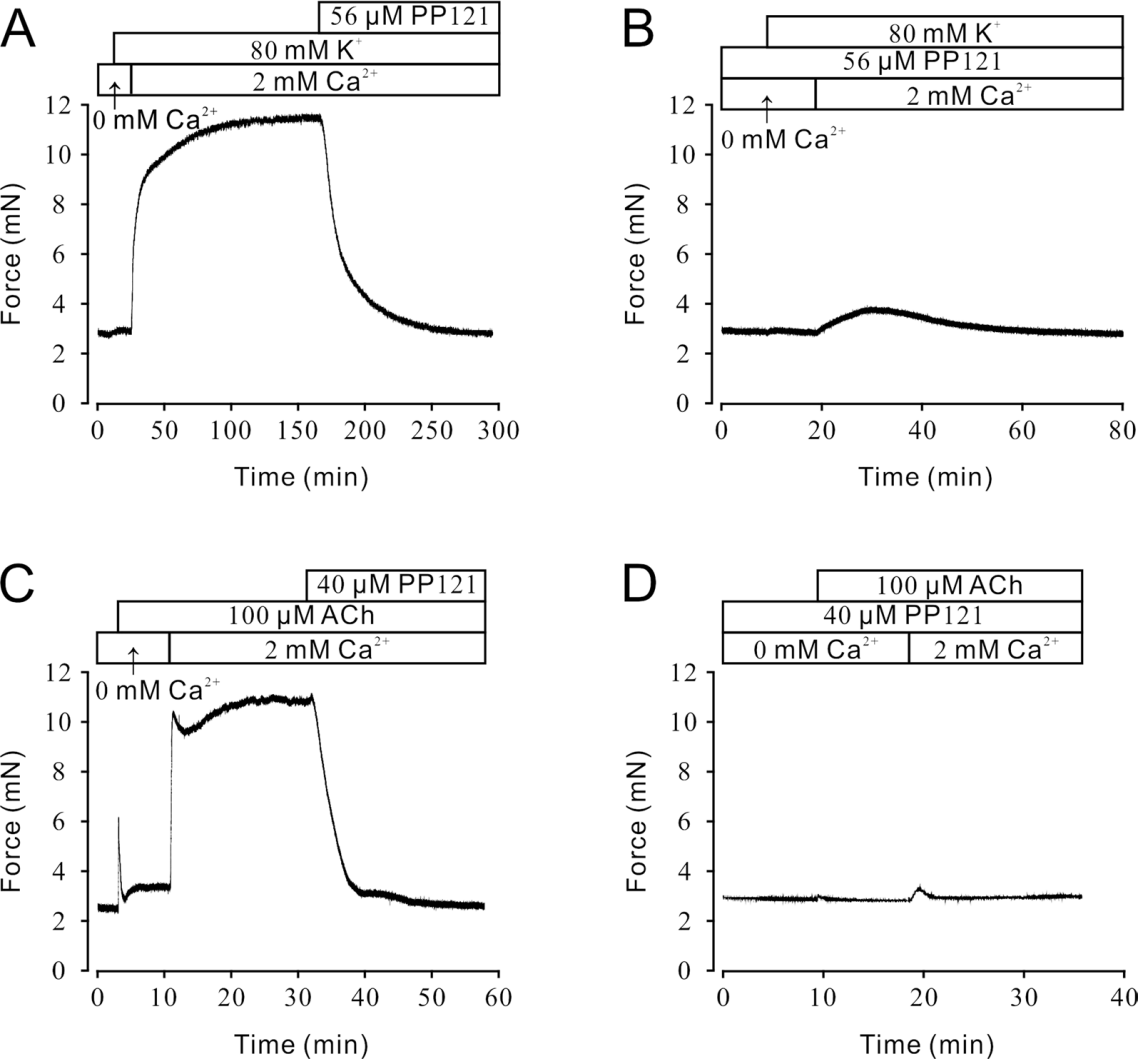
PP121 inhibited high K^+^ or ACh-induced extracellular calcium influx. **A** Under 0 mM calcium conditions, 80 mM K^+^ failed to evoke the contraction of mTRs. When [Ca]^2+^ was restored to 2 mM, 80 mM K^+^-induced steady precontraction of mTRs, which could be completely relaxed by 56 µM PP121 (n = 6/6 mice). **B** In the presence of 56 µM PP121, 80 mM K^+^ failed to induce precontraction on mTRs during 0 to 2 mM Ca^2+^ restoration (n = 6/6 mice). **C** Under the 0 mM calcium condition, 100 µM ACh induced a transient and sharp contraction. When 2 mM [Ca]^2+^ was restored, 100 µM ACh induced a steady contraction, which could be eliminated by 40 µM PP121 (n = 6/6 mice). **D** In the presence of 40 µM PP121, 100 μM ACh failed to induce precontraction on mTRs during 0 to 2 mM Ca^2+^ restoration (n = 6/6 mice)

For analysis of the role of calcium in PP121-induced relaxation on precontracted mTRs, 80 mM K^+^ or 100 μM ACh was employed to evoke contraction under Ca^2+^-free conditions or 2 mM Ca^2+^ conditions. As a result, 80 mM K^+^ rapidly evoked a steady contraction when the calcium in the solution was switched from 0 to 2 mM, and the contraction was completely erased by 56 μM PP121 (Fig. 2A). In contrast, in the presence of 56 μM PP121, 80 mM K^+^ failed to induce contraction when calcium was restored (Fig. 2B). A similar response was observed for 100 μM ACh-induced precontraction. In 0 mM Ca^2+^ solution, 100 μM ACh triggered a sharp and transient contraction, indicating the release of internal calcium. When extracellular calcium was restored, 100 μM ACh induced a steady contraction, which could be completely abolished by 40 μM PP121 (Fig. 2C). Furthermore, 100 μM ACh failed to evoke a contraction in the presence of 40 μM PP121 when the concentration of calcium was restored from 0 to 2 mM. Thus, calcium played an important role in PP121-induced relaxation.

### PP121 switched ion channels

**Fig. 3:**
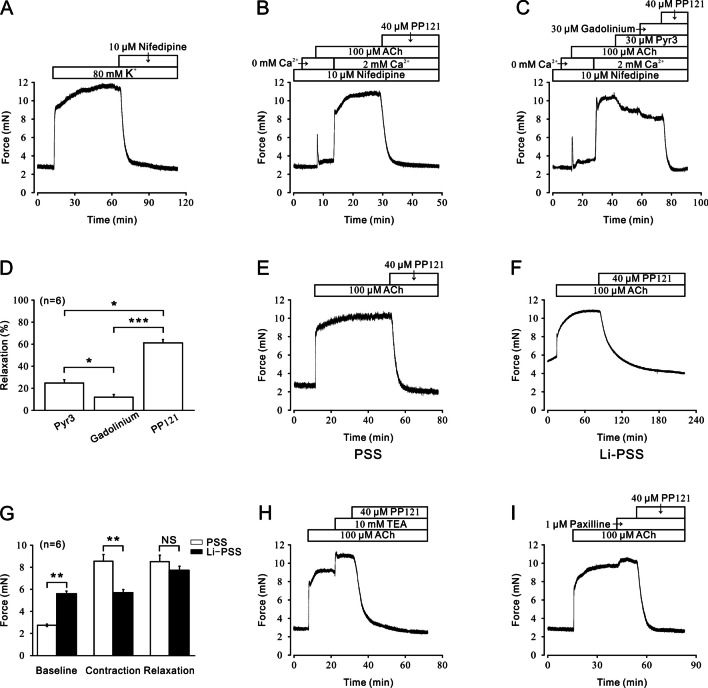
PP121 affected L-VDCCs, NSCCs, NCXs and potassium channels. **A** Treatment with 10 μM nifedipine, an L-VDCC-specific blocker, completely relaxed 80 mM K^+^-induced precontraction (n = 6/6 mice). **B** In the presence of 10 μM nifedipine, 100 µM ACh-induced precontraction during 2 mM calcium restoration was completely eliminated by 40 µM PP121 (n = 6/6 mice). **C** In the presence of 10 μM nifedipine, ACh-induced precontraction in 2 mM Ca^2+^ restoration was sequentially inhibited by 30 μM Pyr3, 30 μM gadolinium, and 40 μM PP121, while the average relaxant percentages of Pyr3, gadolinium, and PP121 are shown in (**D**) (n = 6/6 mice). **E**, **F** PP121 (40 μM) inhibited 100 μM ACh-induced precontraction in PSS or Li-PSS solution. **G** The bar graph shows the significant differences between the net forces of the baseline, contraction, and relaxation (n = 6/6 mice). **H**, **I** Treatment with 10 mM TEA or 1 μM PAX enhanced 100 μM ACh-evoked precontraction. The addition of 40 μM PP121 completely relaxed the precontraction (n = 6/6 mice)

For further analysis of the underlying mechanism of PP121-evoked relaxation on precontracted mTRs, several channel antagonists were applied to identify the involvement of ion channels. As shown in Fig. 3A, nifedipine, a specific L-VDCC blocker, could relax 80 mM K^+^-induced precontraction, which indicated that L-VDCCs might participate in this process. In the presence of nifedipine, 100 µM ACh still evoked a steady contraction that could be completely eliminated by 40 µM PP121 when calcium was restored (Fig. 3B). This result indicated that more ion channels were involved in PP121-induced relaxation. As shown in Fig. 3C, D, L-VDCC was excluded with 10 µM nifedipine. Then, the 100 µM ACh-induced precontraction could be partially blocked by 30 µM Pyr3, 30 µM gadolinium, and 40 µM PP121 sequentially, which indicated that NSCCs, especially TRPC3, were involved in ACh-induced precontraction.

In addition to L-VDCCs and NSCCs, other calcium channels that might participate in PP121-induced relaxation were investigated. As shown in Fig. 3E, under the condition of normal PSS including sodium, ACh induced a steady contraction with a significantly lower basal tone than that under Li-PSS conditions (Fig. 3F), which indicated that extracellular sodium is necessary for maintaining calcium mobilization. Without extracellular sodium, NCX was reversed, and then, the intracellular sodium was transported out and extracellular calcium was transported in, which led to the net contractile force induced by ACh-evoked Ca^2+^ influx in Li-PSS being significantly smaller than that in PSS (Fig. 3G). In addition to 40 µM PP121, 100 µM ACh-induced precontraction could be relaxed even lower than the basal tone under Li-PSS conditions, which indicated that NCX was reversed and drove intracellular Ca^2+^ out. These results indicated that NCX might also participate in PP121-induced relaxation.

Furthermore, the role of potassium channels in PP121-induced relaxation was explored (Fig. 3H, I). As shown in Fig. 3H, 10 mM tetraethylammonium chloride (TEA-Cl), an antagonist of K^+^ channels, could significantly enhance 100 μM ACh-evoked contraction, which indicated that blockade of K^+^ channels could strengthen mTRs contraction. Subsequently, contraction was blocked by 40 µM PP121. To further verify the involvement of the BK channel, which is a typical potassium channel in PP121-induced relaxation, we used PAX, a specific inhibitor of BK channels, as shown in Fig. 3I. The 100 μM ACh-induced contraction could be enhanced by 1 μM PAX. Subsequently, the contraction was reversed with the addition of 40 µM PP121.

Thus, L-VDCCs, NSCCS, TRPCs, NCXs, and K^+^ channels might be involved in PP121-induced relaxation.

### PP121 inhibited ion channel currents

**Fig. 4:**
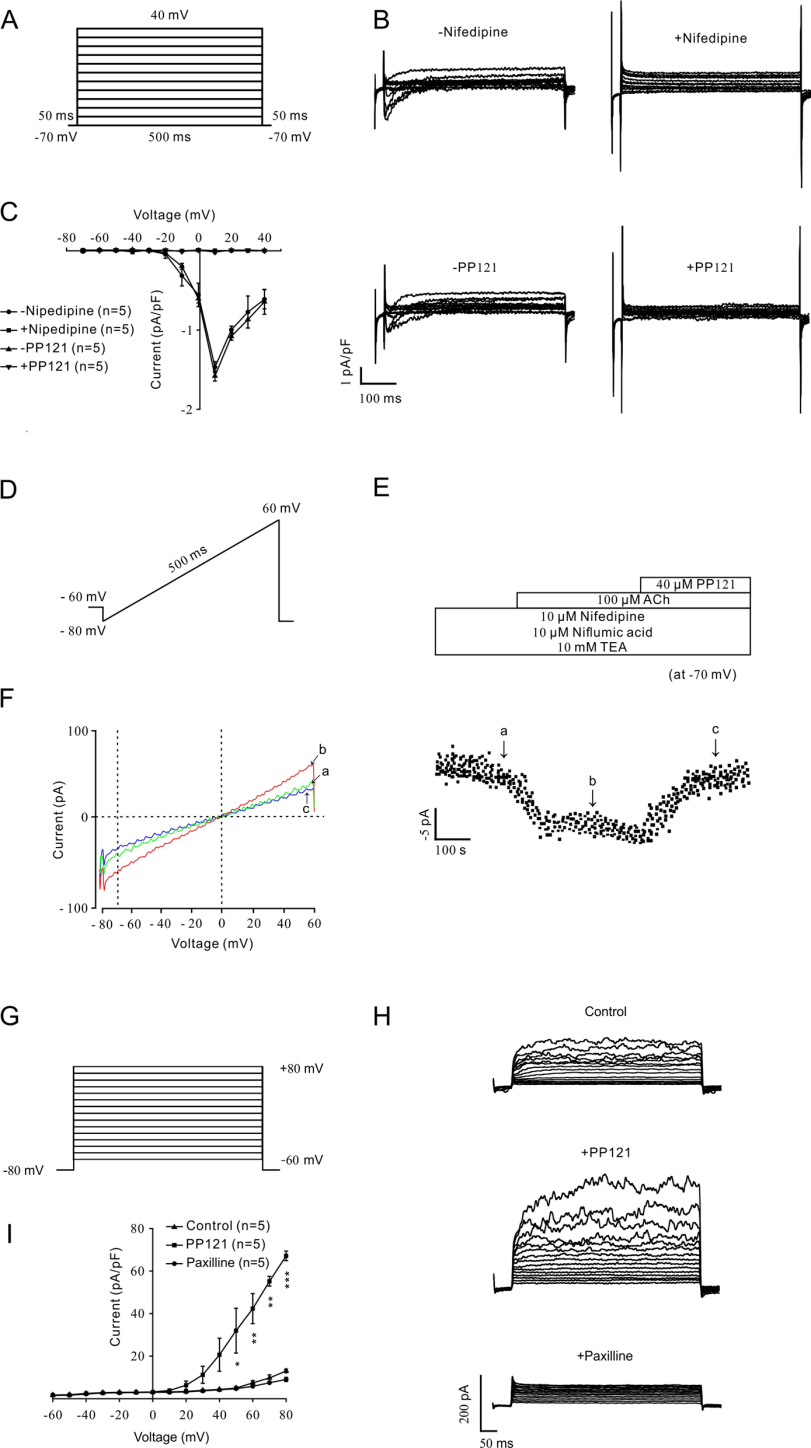
PP121 switched the L-VDCC, NSCC and BK^+^ currents. **A** L-VDCC currents were triggered under a stepped voltage ranging from -70 to + 40 mV in 10 mV increments every 50 ms. **B** L-VDCC currents could be eliminated by 10 μM nifedipine or 56 µM PP121. **C** The current-voltage curve is presented (n = 5/5 mice). **D** NSCC currents were recorded with a 500 ms ramp from − 80 to + 60 mV in 500 ms. **E** 10 μM nifedipine, 10 μM NA and 10 mM TEA were employed to isolate NSCC currents. Then, 100 μM ACh-induced NSCC currents were inhibited by 40 μM PP121 (n = 5/5 mice). The data at − 70 mV were used to plot current-time traces. **F** The net ramp currents at times a, b, and c from − 80 to + 60 mV. **G** BK currents were recorded under ramp voltages ranging from − 80 to + 80 mV at 10 mV increments. **H** BK currents could be erased by a specific blocker, 1 μM PAX, while 40 μM PP121 strengthened the currents. **I** A current-voltage curve was constructed based on the results of 5 cells from 5 mice. *p < 0.05; **p < 0.01; ***p < 0.001

To further identify the involvement of ion channels in PP121-induced relaxation, we measured the L-VDCC currents, NSCC currents and BK channel currents on single mASMCs (Fig. 4). As shown in Fig. 4A, L-VDCC currents were recorded under a stepped voltage ranging from -70 to + 40 mV in 10 mV increments every 50 ms. Then, nifedipine was employed to confirm the record of L-VDCC currents (Fig. 4B, top). Similar L-VDCC currents were also completely erased by 56 µM PP121 (Fig. 3B, bottom). As indicated in the current–voltage (*I-V*) curve of L-VDCCs, PP121 could inhibit L-VDCC currents (Fig. 4C).

For analysis of the effect of PP121 on NSCC currents, whole-cell currents were recorded under a ramp voltage from − 80 mV to + 60 mV in 500 ms (Fig. 4D). Nifedipine (10 μM), 10 μM NA and 10 mM TEA were employed to block L-VDCC currents, Cl^−^ channel currents and K^+^ channel currents, respectively. The isolated NSCC current plots at − 70 mV are presented in Fig. 4E. We discovered that the isolated NSCC currents could be completely blocked by 40 µM PP121. Three representative ramp current traces at time points a, b, and c are shown in Fig. 4F.

For confirmation of the involvement of BK channels in PP121-induced relaxation, BK currents were recorded under voltages ranging from -80 mV to + 80 mV in 10 mV increments (Fig. 4G). We discovered that recorded currents (Fig. 4H, upper) could be completely blocked by 1 μM PAX, a special inhibitor of BK channels (Fig. 4H, bottom), which indicated that BK currents were successfully recorded. With the addition of 40 µM PP121, the BK currents were significantly increased (Fig. 4H, middle). The current–voltage curve is shown in Fig. 4I. These results suggested that PP121 could erase L-VDCC currents and NSCC currents and strengthen BK currents.

### PP121 relieved pathological changes in asthmatic mice

**Fig. 5:**
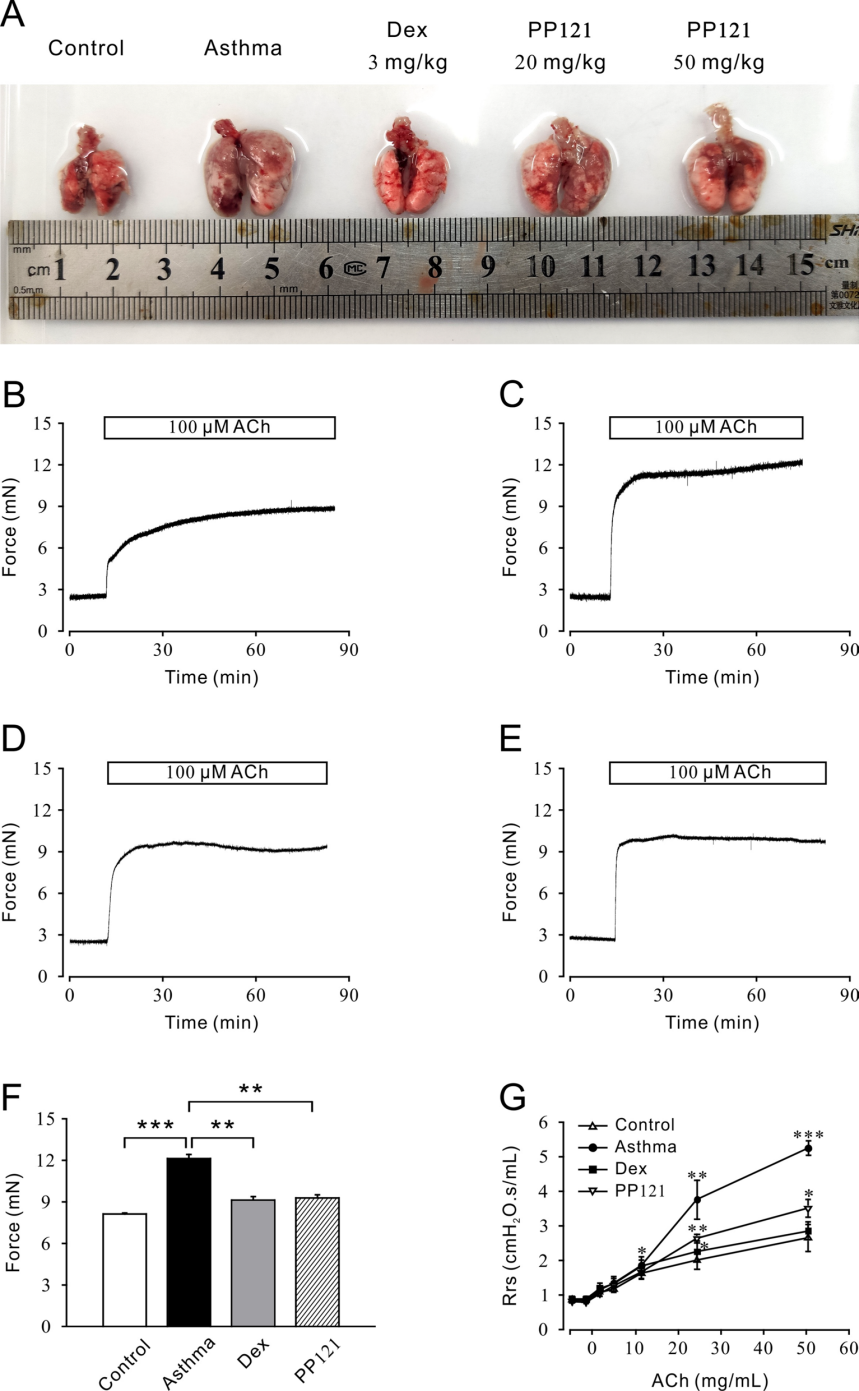
PP121 relieved lung swelling and airway hyperresponsiveness in asthmatic mice. **A** Representative pictures of isolated lungs from the control, asthma, Dex (3 mg/kg) and PP121 groups (20 mg/kg and 50 mg/kg). **B**–**E** ACh (100 μM) induced stable contractions on mTRs isolated from the control, asthma, Dex and PP121 groups. **F** The bar graph shows that the 100 μM ACh-induced contractile forces in the asthma mouse model were significantly higher than those in the control, Dex and PP121 groups (n = 5/5 mice). **G** Gradually added ACh induced significantly higher dose-dependent contractile Rrs curves in asthma than those of the control, Dex and PP121 groups (n = 6/6 mice). *p < 0.05; **p < 0.01; ***p < 0.001

Before performing the in vivo experiment, we explored the effect of PP121 on cell viability in human bronchial epithelial cells. The MTT assay showed that PP121 had no effect on the viability of 16HBE cells after 24 h of incubation at low concentrations (3.16 μM), medium concentrations (10 μM) and high concentrations (56.23 μM) (Additional file 1: Fig. S1F). After 48 h of incubation, 56.23 μM PP121 inhibited cell viability in 16HBE cells, while the low concentration and medium concentration had no effect on cell viability (Additional file 1: Fig. S1G). These results indicated that PP121 had limited harm on cell viability. To further explore the relaxant characteristics of PP121 in asthma treatment, we established an asthma mouse model via OVA stimulation. PP121 (20 mg/kg or 50 mg/kg) was employed to treat the asthma mouse model. The positive control was 3 mg/kg Dex. As shown in Fig. 5A, the isolated trachea and lung from the asthma group were visibly larger than those from the control group. Moreover, the enlargement and swelling were obviously inhibited in the Dex group (3 mg/kg) and PP121 group (20 mg/kg, 50 mg/kg). These results indicated that PP121 could reverse the typical pathological changes of asthma, and the 50 mg/kg PP121 group was chosen for further tension measurement. ACh (100 μM) was applied to induce contraction on isolated mTRs from the control group, asthma group, PP121 group and Dex group (Fig. 5B–E). The statistical graph is shown in Fig. 5F. We discovered that 100 μM ACh induced a significant contraction of mTRs isolated from the asthma group (Fig. 5C) compared with that of the control group (Fig. 5B), while the 100 μM ACh-induced contraction in the Dex group (Fig. 5D) or PP121 group (Fig. 5E) was much lower than that in the asthma group. These results confirmed that the asthma model was successfully established and that PP121 treatment could effectively relieve airway hypertension in vitro. Next, airway hyperresponsiveness was detected in vivo. As shown in Fig. 5G, aerosolized ACh (0, 3.125, 6.25, 12.5, 25 and 50 mg/mL)-induced gradually increased contraction could be statistically inhibited by 50 mg/kg PP121 and 3 mg/kg Dex. These results indicated that PP121 treatment could relieve mouse airway hypertension in vivo.

### PP121 relieved systematic inflammation and mucus secretion in asthmatic mice

**Fig. 6:**
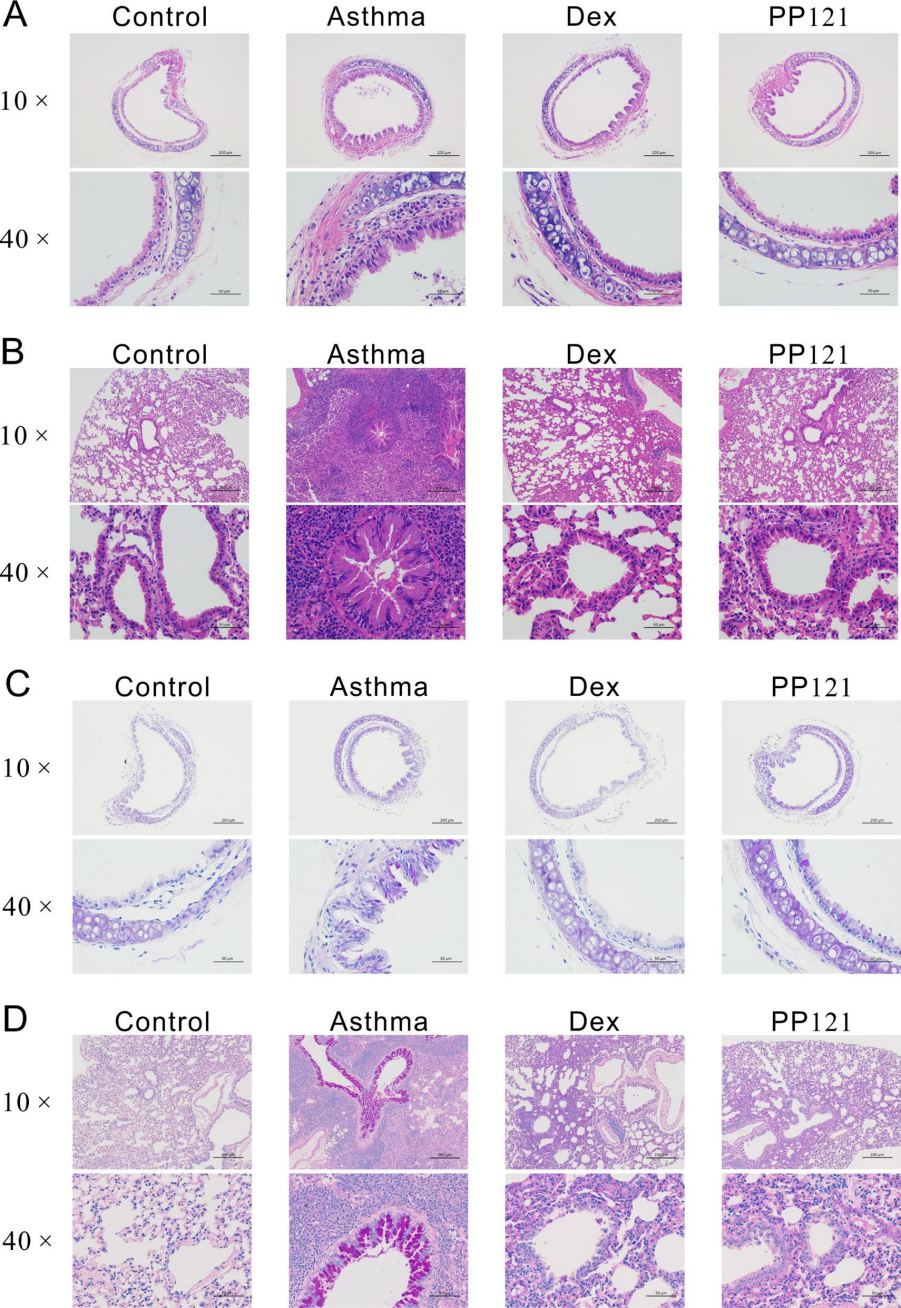
PP121 relieved respiratory inflammation and secretion in asthmatic mice. **A**, **B** Representative H&E staining images of tracheae or lungs derived from the control, asthma, Dex and PP121 groups (n = 3 slides/3 mice). **C**, **D** Representative PAS staining images of tracheae or lungs obtained from the control, asthma, Dex and PP121 groups (n = 3 slides/3 mice)

For analysis of the potential effect of PP121 on asthma, isolated trachea and lung specimens from the control, asthma, Dex and PP121 groups were stained and analyzed (Fig. 6). H&E staining revealed obvious changes in pulmonary structure. As shown in Fig. 6A, the tracheal ring in the asthma group was narrower than that in the control group. The ciliated epithelium was thickened and partly lost. PP121 significantly reversed the thickening of the tracheal ring and rebuilt the lost ciliated epithelium. Dex was applied as a positive control. Similar bronchial narrowing and abnormal proliferation were also observed in asthmatic lung sections (Fig. 6B). In contrast, the PP121 treatment relieved bronchial narrowing and reduced cell proliferation. These results indicated that PP121 treatment could rebuild the trachea and lung structure in an asthmatic mouse model.

Furthermore, PAS staining was performed to detect the secreted mucin in trachea and lung specimens. In the asthma group, the positive signal of mucin was increased in the tracheal ciliated epithelium compared with that of the control group (Fig. 6C). After PP121 treatment, the abnormal secretion of mucin was significantly reduced. Abnormal mucin secretion was also observed in lung specimens (Fig. 6D). Various PAS-labeled mucins in bronchial lumens indicate that the abnormal proliferation of goblet cells was increased in the asthma group. Moreover, PP121 treatment resulted in a significant reduction in goblet cells, suggesting that PP121 could reduce mucus secretion in the asthmatic group. All these results indicated that PP121 could relieve asthmatic conditions such as airway thickening, loss of ciliated epithelium and mucus secretion in vivo.

### PP121 decreased MAPK/Akt signaling pathways in asthmatic mice

**Fig. 7:**
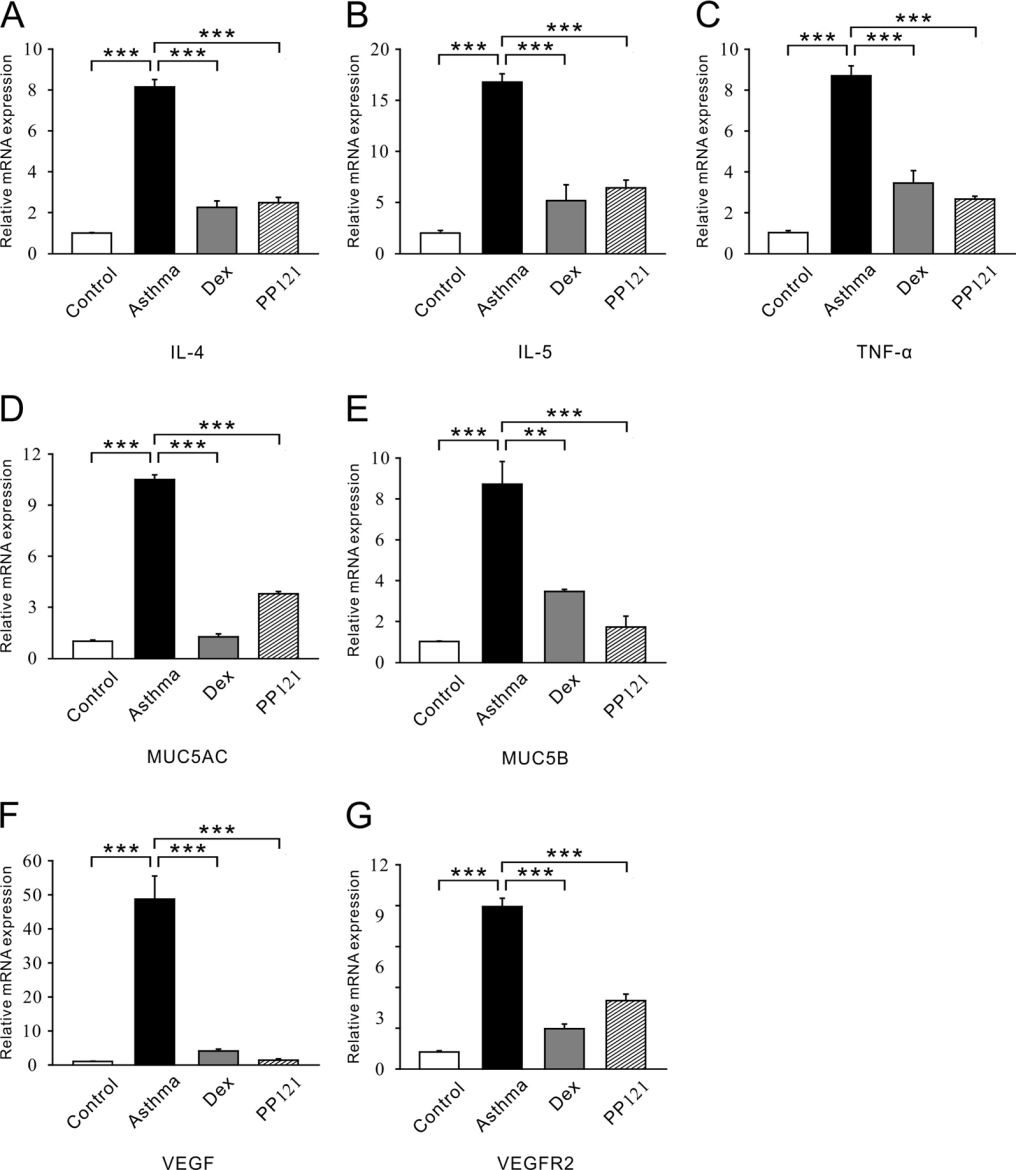
PP121 decreased the mRNA expression levels of inflammatory cytokines, mucins and RTKs in lung tissues. **A**–**C** The bar graph shows the different expression levels of IL-4, IL-5, TNF-α, MUC5AC, MUC5B, VEGF and VEGFR2 in the control, asthma, Dex and PP121 groups. **p < 0.01; ***p < 0.001

RT‒PCR was employed to detect the expression levels of inflammatory cytokines and mucins. We found that the mRNA expression levels of several inflammatory factors, such as IL-4, IL-5 and TNF-α, were significantly lower in the PP121 group than in the asthma group (Fig. 7A–C). MUC5AC and MUC5B, two key factors of mucins, were downregulated in the PP121 group (Fig. 7D, E). VEGF and VEGFR2 were also downregulated after PP121 treatment (Fig. 7F, G).

**Fig. 8:**
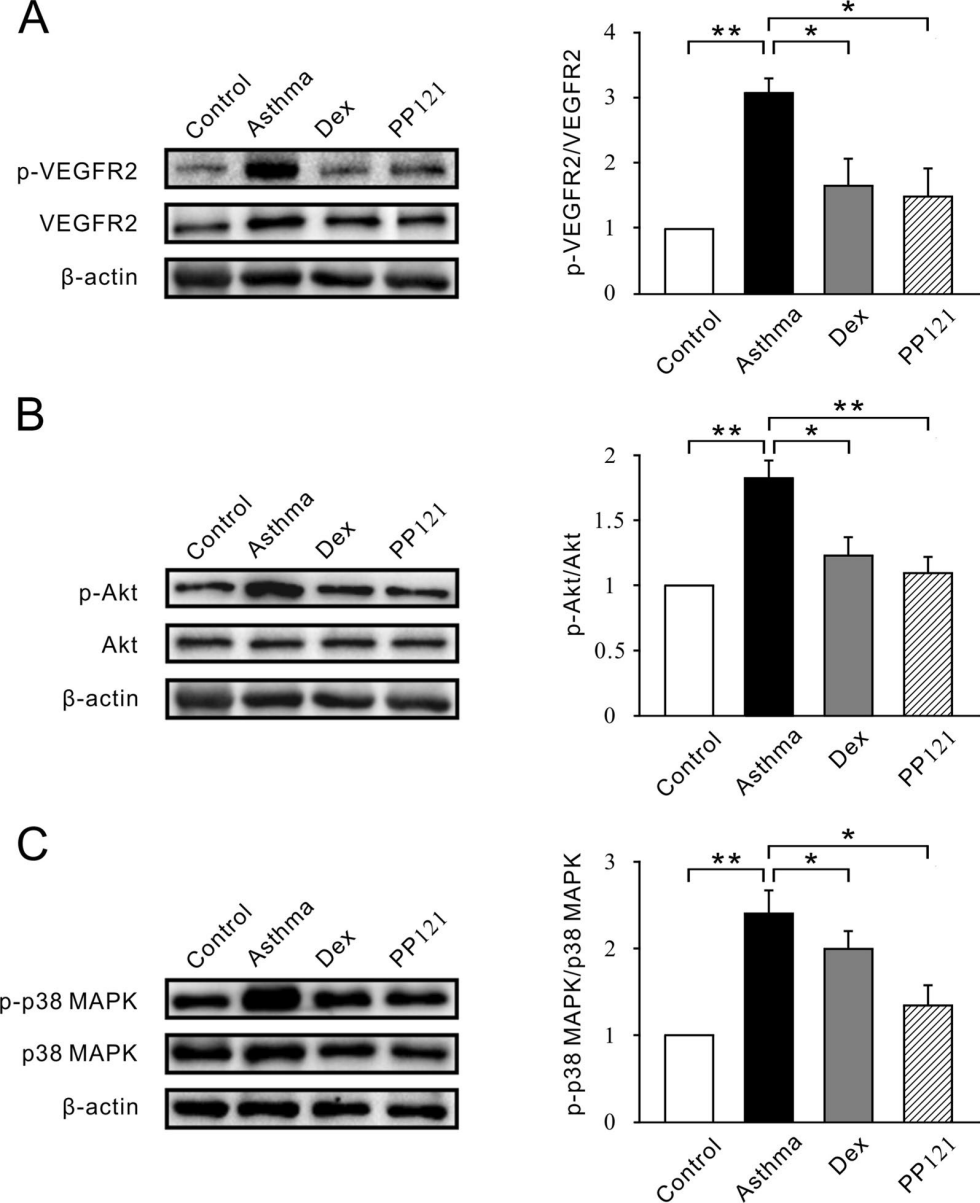
PP121 inhibited VEGF and MAPK/Akt signaling pathways in lung tissues. **A**–**C** The western blot band (left panel) and bar graph (right panel) show the ratio of p-VEGR2 to VEGFR2, p-Akt to Akt and p-p38 MAPK to p38 MAPK in the control, asthma, Dex and PP121 groups, respectively. *p < 0.05; **p < 0.01

The phosphorylation of VEGFR2, Akt and p38 MAPK was further investigated. We discovered that the ratios of p-VEGR2 to VEGFR2, p-Akt to Akt and p-p38 MAPK to p38 MAPK were elevated in the asthma group compared with the control group (Fig. 8A–C). Then, the phosphorylation of VEGFR2, Akt and p38 MAPK was found to be decreased in the PP121 group and Dex group. Taken together, these data indicated that PP121 affects the MAPK/Akt signaling pathway.

## Discussion

Asthma is a systematic respiratory disease wherein airway hyperresponsiveness, systematic inflammation and airway remodeling are the main symptoms. Tyrosine kinases and phosphoinositide kinases play crucial roles in molecular and physiological mechanisms orchestrating asthma pathophysiology and represent potential therapeutic targets (Yoo et al. 2017; Guntur and Reinero 2012). Various tyrosine kinase and phosphoinositide kinase pathways contribute to aspects of airway hyperresponsiveness, systematic inflammation and airway remodeling. Recently, studies of asthma treatments have focused on TKIs and PKIs.

PP121 is a dual inhibitor of tyrosine kinase and phosphoinositide kinases, and its antitumor properties have been identified in esophageal cancer, prostate cancer and hepatocellular carcinoma (Peng et al. 2015; Bello, et al. 2021; Zhai et al. 2021). Recently, PP121 has been shown to exert an antitumorigenic effect in primary and metastatic non-small cell lung cancers (NSCLC) (Quick 2023). However, the role of PP121 in asthma is still unclear. Considering the crucial role of tyrosine kinase and phosphoinositide kinase pathways in asthma, we aimed to investigate the potential efficacy of PP121 for asthma treatment in the current study.

We explored the therapeutic effect of PP121 on typical asthma signs, especially abnormal airway contraction and systemic inflammation. Primarily, we found that PP121 could significantly relax isolated mTRs precontracted by high K^+^ or ACh, which confirmed the anticontractile property of PP121. In the respiratory tract, a series of ion channels are expressed in airway cells and control the balance of intracellular Ca^2+^ and extracellular Ca^2+^ concentrations. Abnormal ion channel switching ultimately activates the pathogenesis of asthma (Valverde et al. 2011; Muller, et al. 2022). The exploration of participating ion channels revealed that L-VDLCCs, NSCCs, NCXs and potassium channels were regulated and that the channel currents were altered during the process of PP121-induced relaxation. The possible explanation was that the addition of PP121 could block L-VDCCs and NSCCs, switch NCXs, and activate BK channels to inhibit Ca^2+^ influx and then induce airway relaxation.

Subsequently, an asthmatic mouse model with PP121 treatment was employed to detect the therapeutic efficacy of PP121 in vivo. The observed lung enlargement and abnormal contraction indicated that the animal models were successfully established. Further Rrs measurements showed that PP121 could attenuate ACh-induced airway hyperresponsiveness. Histological analysis of lung specimens showed that typical signs of asthma were obviously relieved with the addition of PP121. Airway narrowing was significantly relieved, which was consistent with the relaxant effect of PP121 on isolated mTRs. PP121 is a potential inhibitor targeting VEGFR2 in thyroid tumors (Dunna et al. 2015). In our asthma mouse model, the expression levels of VEGF and VEGFR2, which play important roles in airway remodeling through their effect on inflammation and angiogenesis (Smith 2014; Huang et al. 2016), were decreased, which indicated that VEGF and VEGFR2 were also potential targets of PP121 in asthma. Mucus secretion was also reduced, and the downregulation of two key mucins, MUC5AC and MUC5B, which are integral components of mucus dysfunction in asthma (Bonser and Erle 2017), was observed in the PP121-treated mice. Furthermore, systematic inflammation was significantly relieved in the asthmatic mice treated with PP121. TNF-α (Berry et al. 2007), IL-4 (Massey and Suphioglu 2021) and IL-5 (Principe et al. 2021), which participate in the inflammatory formation of asthmatic lungs, were significantly reduced.

The MAPK/Akt pathway is an important signaling pathway in asthma formation that participates in airway remodeling and inflammation (Liu et al. 2023; Lyu et al. 2022; Jin et al. 2023). To further identify the possible involved signaling pathway, we determined the expression levels of MAPK and Akt, which are important components of the MAPK/Akt pathway (Ma et al. 2016; Bai et al. 2022; Liu et al. 2015). We discovered that PP121 reduced the phosphorylation of MAPK and Akt. This result indicated the participation of MAPK/Akt signaling in the PP121-induced attenuation of asthma. Thus, PP121 could relieve asthmatic conditions via downregulation of proangiogenic factors (VEGF and VEGFR2), key mucins (MUC5AC and MUC5B), and inflammatory factors (TNF-α, IL-4 and IL-5) and inhibition of the MAPK/Akt signaling pathway.

Through our research, the anticontractile and anti-inflammatory characteristics of PP121 were confirmed in asthmatic mice, and the underlying molecular mechanism was explored. Hence, PP121 could be considered a potential candidate drug to relieve asthma characteristics, including airway remodeling, airway hypersecretion and systematic inflammation. However, the efficacy and safety of PP121 need to be further confirmed via necessary clinical trials.

## Conclusions

Our studies suggested that PP121, a novel dual inhibitor of tyrosine and phosphoinositide kinases, could relax precontracted mTRs via the alteration of calcium mobilization and blockage of certain ion channels, including L-VDCCs, NSCCs, NCXs and BK channels, in vitro. Furthermore, animal experiments revealed that PP121 could effectively relieve typical asthma conditions, including airway hyperresponsiveness, mucus secretion, airway remodeling and systematic inflammation via the MAPK/Akt signaling pathway. Thus, the therapeutic role of PP121 in asthma treatment as a potential anticontractile and anti-inflammatory drug was primarily confirmed.

### Supplementary Information

**Additional file 1: Figure S1.** PP121 had limited harm to tissue bioactivity. **A** PP121 (56 µM) had no effect on the basal tone of mTRs (n = 7/7 mice). **B**, **C** Precontraction induced by 80 mM K^+^ could be inhibited by 56 μM PP121. After washout, a similar contraction was evoked by 80 mM K^+^ (n = 6/6 mice). **D**, **E** The 100 μM ACh-induced precontraction could be inhibited by 40 μM PP121. After washout, a similar contraction was evoked by 100 μM ACh (n = 6/6 mice). **F**, **G** PP121 had a limited effect on cell viability in 16HBE cells at 24 or 48 h.

## Data Availability

All data generated and analyzed in this study are available upon reasonable request from the corresponding author.

## Abbreviations

16HBE: Human bronchial epithelial cell line
ACh: Acetylcholine chloride
BK channel: Large-conductance Ca^2+^-activated K^+^ channel
BSA: Bovine serum albumin
Dex: Dexamethsone
DMSO: Dimethyl sulfoxide
DTT: Dithiothreitol
HBSS: Hanks’ balanced salt solution
H&E: Hematoxylin and eosin
HRP: Horseradish peroxidase
IC50: Half-maximal inhibitory concentration
IL-4: Interleukin 4
IL-5: Interleukin 5
IP: Intraperitoneally
L-VDCCs: L-type voltage-dependent Ca^2+^ channels
MAPK: Mitogen-activated protein kinase
mASMCs: Mouse airway smooth muscle cells
mTOR: Mammalian target of rapamycin
mTRs: Mouse tracheal rings
MTT: 3-(4,5-Dimethylthiazol-2-yl)-2,5-diphenyltetrazolium bromide
MUC5AC: Mucin 5 subtype AC
MUC5B: Mucin 5 subtype B
NA: Niflumic acid
NC: Nitrocellulose
NCXs: Na^+^/Ca^2+^ exchangers
NFκB: Nuclear factor kappa-B
NSCCs: Nonselective cation channels
NSCLC: Non-small cell lung cancers
OVA: Ovalbumin
PAS: Periodic acid-Schiff
PAX: Paxilline
PFA: Paraformaldehyde
PI3K: Phosphatidylinositol-3-kinase
PKIs: Phosphoinositide kinase inhibitors
PSS: Physiological salts solution
Pyr3: Pyrazole 3
RTKs: Receptor tyrosine kinases
Rrs: Respiratory system resistance
SD: Standard deviation
SDS-PAGE: Sodium dodecyl sulfate–polyacrylamide gel electrophoresis
SPF: Specific pathogen-free
TBST: Tris-buffered saline Tween-20
TEA-Cl: Tetraethylammonium chloride
TKIs: Tyrosine kinase inhibitors
TNF-α: Tumor necrosis factor alpha
TRPCs: Transient receptor potential channels
VEGF: Vascular endothelial growth factor
VEGFR2: Vascular endothelial growth factor receptor 2
