# Supplementary figures and images for: PP121, a dual inhibitor of tyrosine and phosphoinositide kinases, relieves airway hyperresponsiveness, mucus hypersecretion and inflammation in a murine asthma model
Source: Mol Med. 2023 Nov 7;29:154. doi: 10.1186/s10020-023-00748-w (PMC10629066; doi:10.1186/s10020-023-00748-w)

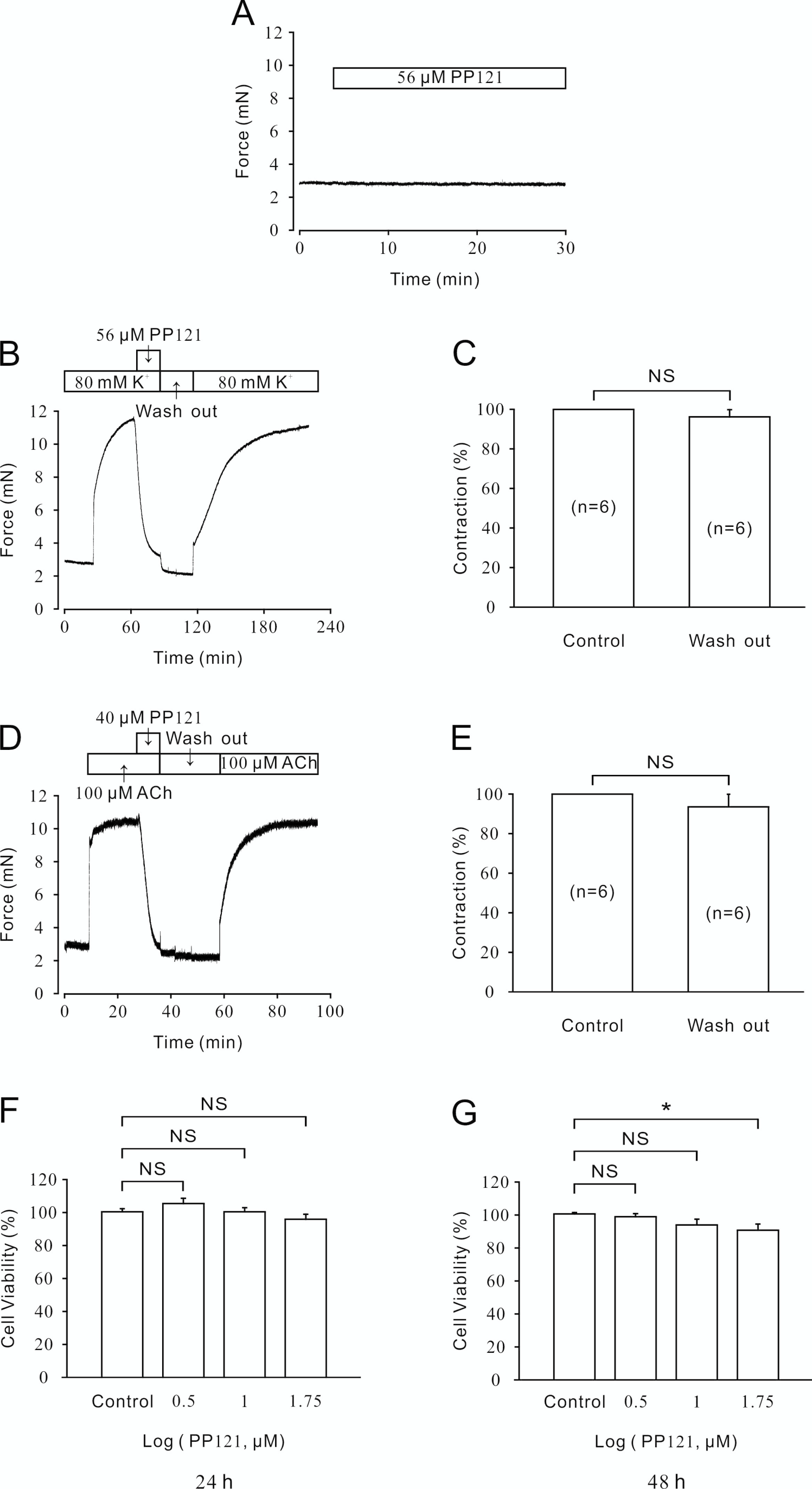

Supplement: Supplementary file 1 — Additional file 1: Figure S1. PP121 had limited harm to tissue bioactivity. A PP121 (56 µM) had no effect on the basal tone of mTRs (n = 7/7 mice). B, C Precontraction induced by 80 mM K+ could be inhibited by 56 μM PP121. After washout, a similar contraction was evoked by 80 mM K+ (n = 6/6 mice). D, E The 100 μM ACh-induced precontraction could be inhibited by 40 μM PP121. After washout, a similar contraction was evoked by 100 μM ACh (n = 6/6 mice). F, G PP121 had a limited effect on cell viability in 16HBE cells at 24 or 48 h. [file 10020_2023_748_MOESM1_ESM.pdf]
